# Integrated secure messaging to enhance medical education: a mixed methods study

**DOI:** 10.1186/s12909-022-03637-8

**Published:** 2022-07-28

**Authors:** Laura Nichols, Dubert Guerrero, Devendranath Mannuru, Marc D. Basson, Abe E. Sahmoun, Dinesh Bande

**Affiliations:** 1 Sanford Health, 801 N Broadway, Fargo, ND 58103 USA; 2 University of North Dakota School of Medicine and Health Sciences, 1301 N Columbia Rd, Grand Forks, ND 58203 USA; 3 Department of Internal Medicine, University of North Dakota School of Medicine and Health Sciences, 1919 Elm St N, Fargo, ND 58102 USA

**Keywords:** Medical education, Healthcare communication, Secure messaging

## Abstract

**Background:**

Instant messaging applications and texting are useful for educating and communicating with medical students; however, they present patient privacy concerns and do not address the challenge of student inclusion in patient care communication. EMR-integrated secure messaging offers an opportunity to include students on team communication, enhance their medical education, and ensure patient privacy.

**Methods:**

Between July 2019 through March 2020, we performed a mixed method study to evaluate use of EPIC® Secure Chat as a means of enhancing student education and team communication. We promoted use of secure messaging in orientation, performed a pre- and post-rotation survey to assess perceptions of Secure Chat effect on communication, and directly reviewed and categorized messages.

**Results:**

Twenty-four 3rd and 4th year students completed the pre-rotation survey, and 22 completed the post-rotation survey. Twelve (50%) students reported the quality of communication with faculty was either good or very good prior to internal medicine rotation, while 20 (91%) reported this post-rotation (p-value 0.001). There was a similar improvement in communication with ancillary staff. Nineteen (86%) students felt that secure messaging improved their communication with faculty. On message review, threads were frequently logistical, but also often included discussions of patient management.

**Conclusions:**

Students viewed Secure Chat as having a favorable effect on their communication with team members and reported communication on internal medicine to be improved compared to prior rotations. Messages included students on important patient care conversations. Secure messaging offers a novel medium to improve team communication, enhance student education, and maintain patient privacy.

## Background

In educating medical students, physician educators are continually working to balance concerns of adequate medical student training and patient safety and autonomy. The American Medical Association (AMA) code of ethics notes that while “Having contact with patients is essential for training medical students…the obligation to develop the next generation of physicians must be balanced against patients’ freedom to choose from whom they receive treatment” [1]. Additionally, there may be decreased patient satisfaction when medical students participate in patient care [2]. In this context, medical education has become less active and more passive, decreasing student preparedness for practice in residency and beyond [3]. Movement of care from the bedside to the computer has created additional challenges for active student involvement. Frequently ancillary staff such as nursing are unaware of which student is working directly with their patients or the student may not have a device such as a pager to utilize, which makes communication with students more difficult. These barriers necessitate implementing creative ways to balance student participation with patient autonomy and privacy.

While technology may create barriers to direct student involvement, it also presents other opportunities for increasing student engagement. Media such as email, texting and social media have been utilized to improve resident and student knowledge of a variety of subjects from pathology to geriatrics to reading EKGs [4–6]. Additionally, texts are utilized frequently for direct communication with 60-80% of residents and attending physicians in a variety of specialties exchanging text messages related to patient care [7–9]. Reasons cited for utilizing text messages include ease of integration into workflow and communication efficiency and clarity [10–12]. However, media such as text messaging have not been widely used to improve student involvement in direct patient care. Additionally, these platforms may present patient privacy concerns if information is not shared in an appropriately de-identified manner and do not address issues regarding lack of student inclusion in patient care conversations [5, 10, 13–15].

Electronic medical record (EMR)-integrated secure messaging is increasingly being utilized as a tool for improved communication and protection of patient information in health systems. Though there are many secure messaging platforms, there is a paucity of literature outlining best practices on utilization of secure messaging and no literature investigating its role in medical education [16]. EMR-integrated secure messaging was introduced in our hospital system in the United States 1-2 years prior to the current study as an additional method of communicating between physicians and other care team members. Messages sent in the platform are deleted after 14 days and are not part of the legal medical record to allow users to have fluid conversation and the ability to review recent messages without overwhelming the EMR with stored data from dated conversations. The rollout of this platform offered an opportunity to include students in direct patient care conversations and utilize it for educational purposes during their internal medicine rotation. Increasing utilization of technology in medical care compels us to deliberately incorporate these tools into medical student education to produce physicians better prepared for electronic communication that respects patient privacy. Furthermore, secure messaging offers educators an additional tool for both formal and “bedside” teaching. Given the paucity of existing literature, it was imperative to first investigate current use and then to work toward creating curricula and best practices. Finally, an understanding of student satisfaction with communication and learning methods is important for mindful incorporation of any curriculum. These multi-faceted aims (improved communication, medical education, and student satisfaction) were the impetus behind the creation of our current study. We hypothesized that encouraging students to use integrated secure messaging with their team and clerkship directors would enhance their feelings of satisfaction with the clerkship and involvement in care communications. Additionally, we postulated that secure messaging was already being utilized with students in several ways, including logistical communication and bedside teaching.

## Methods

Between July 2019 through March 2020, we performed a mixed methods study at a single rotation site to investigate 3^rd^ and 4^th^ year medical student perceptions of secure messaging effect on the quality of their internal medicine rotation and student inclusion in patient care communications. The study underwent IRB review at Sanford Health and the University of North Dakota and did not require ongoing IRB surveillance. Thirty-eight students were included based on rotating at our hospital site on a 3^rd^ year clerkship or 4^th^ year acting internship during the study period. Formal informed consent was waived by the Sanford Health and University of North Dakota IRB. Use of Secure Chat was promoted and students were informed of the study during rotation orientation. Students were provided with small tablets with the EMR application to ensure they had secure messaging access without requiring them to use their personal devices, but were allowed to use their own devices if they wished. Given we had a limited number of devices and different sites had different EMR systems, only students from one campus were included in the current study. Rather than compare specific sites, we utilized student perceptions to compare their communication experiences on prior rotations with their experiences on internal medicine given secure messaging was not widely utilized in other departments prior to the implementation of this study. Integrated secure messaging was incorporated directly into the clerkship for communications about case discussions and discharge summary feedback. Message content was reviewed after grades were finalized to assuage potential concern about being evaluated based on message content.

Outcomes were measured primarily based on student perceptions of Secure Chat effect on communication and education experience using a pre- and post-rotation survey using a 5-point Likert scale without specific anchors for questions related to student satisfaction. There were not existing validated questionnaires related to this topic; therefore, questions were created based on input from multiple faculty. For the questions regarding improvement in communication on internal medicine, median (range) and/or mean ± SD were calculated for all the continuous variables. The comparisons of faculty communication pre-rotation/faculty communication post-rotation and ancillary staff communication pre-rotation/ancillary staff communication post-rotation were performed using the non-parametric Wilcoxon signed-rank test or paired t-test. Statistics were performed using SAS (SAS Institute, Cary, NC, USA; Version 9.4 Users Guide). All the statistical tests were two-sided with *p* < 0.05 considered to be significant.

Additional study data was obtained from faculty and residents and review of message threads. Faculty and residents provided qualitative feedback on use of Secure Chat with students through direct conversation and email questionnaires. A direct review of secure messaging threads and categorization of content was also completed to evaluate how secure messaging was used with medical students. Due to lack of existing literature, categories were created based on themes identified during message review and were as follows: logistical communication, daily patient management issues, discharge coordination, admissions, educational, clerkship-related, procedures, and scholarly activity.

## Results

**Fig. 1:**
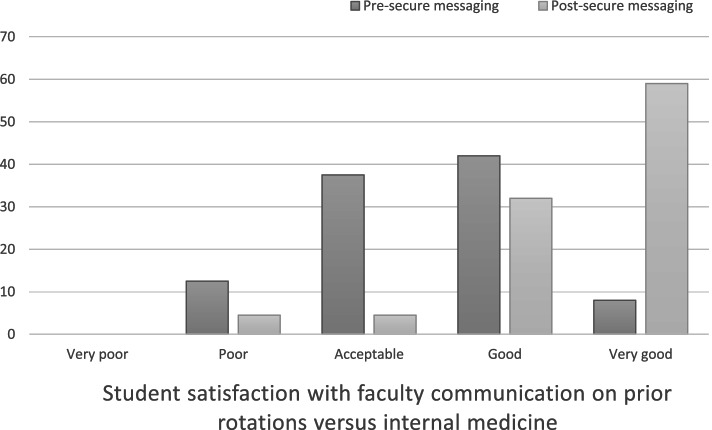
Student satisfaction with faculty communication on internal medicine**.** Graph displays the percentage of students reporting satisfaction with faculty communication on prior rotations (pre-rotation survey) versus communication on their internal medicine rotation (post-rotation survey)

Twenty-four of 38 students completed the pre-rotation survey for a 63% response rate, and 22 of 38 completed the post-rotation survey for a 58% response rate between July 2019 and March 2020. On prior rotations, students reported using direct communication and texting most frequently; whereas, direct communication and secure messaging were most frequently used on internal medicine. Twelve (50%) students reported the quality of communication with faculty was either good or very good prior to internal medicine rotation while 20 (91%) reported this perception at conclusion of the rotation (*p*-value <0.001) (Fig. 1).

**Fig. 2:**
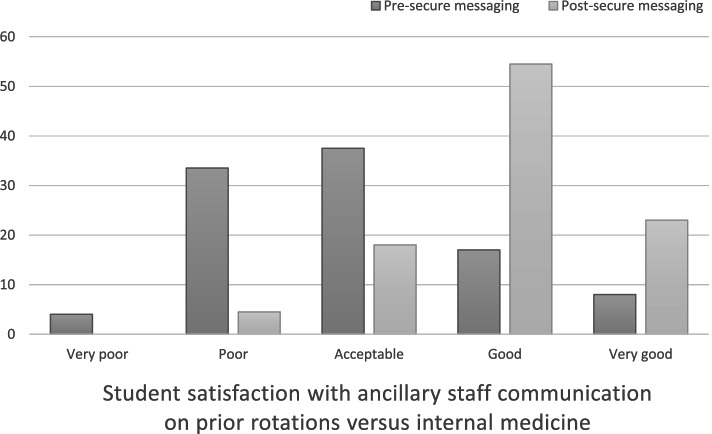
Student satisfaction with ancillary staff communication on internal medicine**.** Graph displays the percentage of students reporting satisfaction with ancillary staff communication on prior rotations (pre-rotation survey) versus communication on their internal medicine rotation (post-rotation survey)

**Fig. 3:**
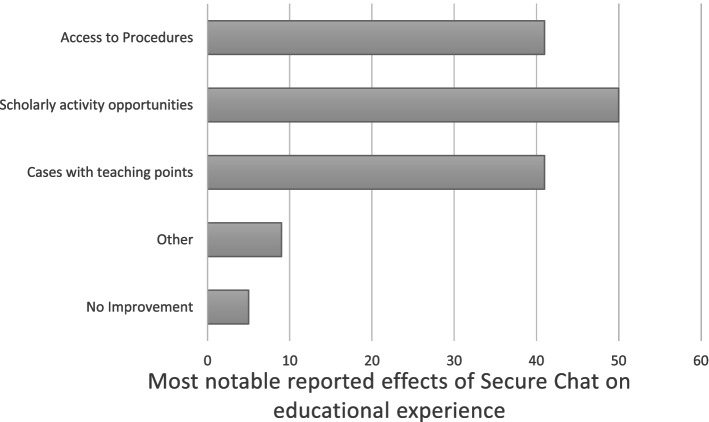
Most notable reported effects of Secure Chat on educational experience. Graph displays the reasons students reported improvement in their educational experience through use of Secure Chat displayed as percentages. More than one selection was allowed. The Other category had two write-ins that reported the ease of communication was the most prominent effect of Secure Chat on the educational experience

Six students (25%) reported good or very good communication with ancillary staff prior to their internal medicine rotation as compared to 17 (77%) in the post-internal medicine rotation survey (*p*-value 0.011) (Fig. 2). When specifically questioned about the effect of secure messaging on communication during the rotation, nineteen (86%) students felt that Secure Chat either somewhat or significantly improved their communication with faculty, and 16 (73%) reported this outcome for their communication with ancillary staff. Twenty-one (95%) students reported that secure messaging resulted in either somewhat or significantly improved ease of communication on the internal medicine rotation, and 17 (77%) felt it grew their educational experience (Fig. 3).

Qualitative feedback was collected from both residents and faculty on the use of secure messaging with students. Consistently, both residents and faculty expressed that it allowed them to better include students on the team, to provide real-time updates on patient care and to create better student ownership of their patients. They also noted that it was a secure space to share patient records, including for scholarship. Barriers that were identified included that some students did not use it as a means of communication consistently, and at times preceptors would not get a timely response from students. Adding students to conversations created some extra work, as it was more difficult to search for students than other members of the care team, but this was overall minimal. Finally, there was concern about potential duty-hour violations when students were receiving Secure Chat messages at home.

**Fig. 4:**
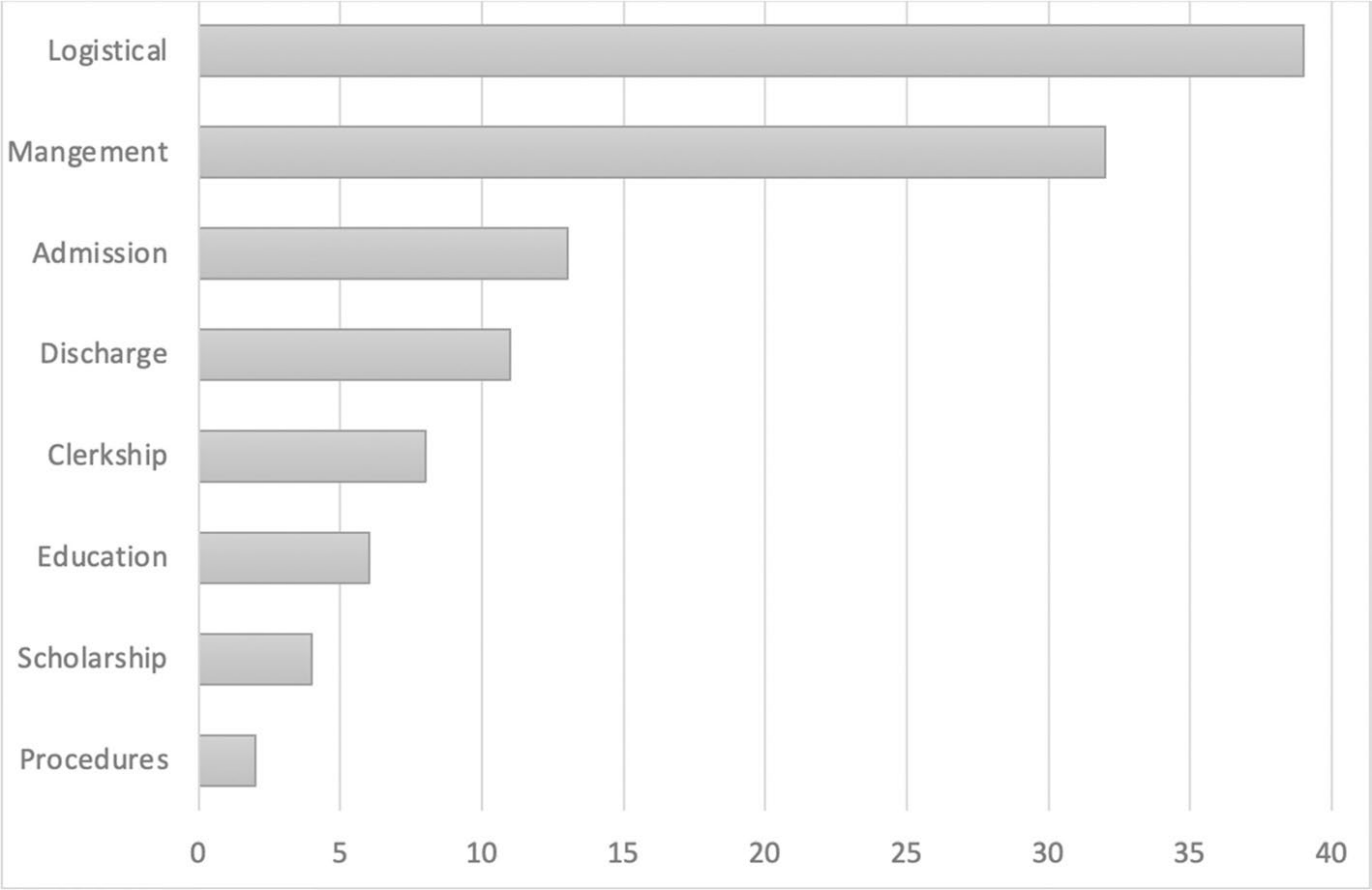
Secure message content by category. Graph displays the percent of the 826 total message threads containing each content category. Note that a single message thread may contain more than one content category

In addition to student perceptions, we reviewed 826 Secure Chat message threads that included 3^rd^ and 4^th^ year medical students to better understand the types of communications that involved students. There was logistical communication in 325 (39%) message threads, which was the most frequent type of communication (Fig. 4).

Specific content generally included meeting times and locations as well as clarifications on note completion and note changes. Daily patient management issues, such as changes in medications, coordination with consulting services, and clarification of orders with nursing were included in 261 message threads (32%). Secure Chats about new admissions (13%) and discharge planning communications (11%) were the next most frequent categories. Educational and clerkship-related communications were less common and included in 8% and 6% of message threads, respectively. Specific educational content included quick teaching points and links to articles pertinent to patient care. Although numbers were too small for adequate statistical power, we observed a trend toward more passive communication with 3^rd^ year students as opposed to 4^th^ year students (Table 1). Third year students tended to be included in conversations primarily meant for others such as logistical communication with case management. They were also frequently passively included in admission communications. Additionally, third year students communicated peer-to-peer regarding procedures. Fourth-year students were involved in more active conversations with consultants, ancillary staff and preceptors as demonstrated in “Management communication.” Educational communications with 3^rd^ or 4^th^ year students included informal feedback, teaching pearls, and conversations regarding medical literature application to patient care.

**Table 1:** Examples of Secure Chat threads. Table displays specific examples of a variety of Secure Chat thread types (logistical, educational, admission communication, etc.)

**Logistical communication**
*“Let me know when get to floors. 622 & 625: Inpt Rehab is viewing for discharge planning. They will let me know if can be accepted today. […] 634: Still no word back from CHI at Oakes. […]”* *“We are going to 663”*
**Admission communication**
*“please admit for nausea, vomiting and [d]iarrhea x 2 days, normal cbc and cmp. elevated lactic acid, influenza was negative, likely viral gastroenteritis. […]. CT scan was done […] normal LFT”* “sounds good”
**Procedure communication**
*“Wanna go to third floor for a pacemaker soon?”* *“Yes!”* *“I’ll come to the lounge before so I’ll meet you down there!”* *“Okay, perfect, I’m just working on notes here”*
**Clerkship feedback**
*“[student name]- Your discharge summary was excellent. I did not have any questions about the hospital course and your listing of the diagnoses and follow ups was good. Additionally, you separated out things by problem. Let me know if you have any questions about this feedback.”*
**Management communication**
*“Hi, [patient’s PCP], just wanted to let you know that [your patient] was admitted for recurrent epistaxis. ENT recommended that we hold Warfarin and Aspirin and they will be following up in clinic tomorrow to remove packing. […] Please resume anticoagulation once the bleeding has stopped [and] let me know if you have any questions.”* *“OK / Thank you for letting me know”*
*“Hi [hematologist/oncologist], [the patient] was admitted for a right radial fracture after a fall at home [and underwent ORIF]. Is it okay to restart [medication]?”* *“OK to re-start [medication]. Is the plan to place oncology consult or is she discharging soon, thanks.”* *“Will check with my attending and let you know whether consult will be needed. Thanks”.* *“Not necessary to place consult if stable, thanks.”*
**Educational communication**
*“[Student], I heard about a great job you did with [Dr. Preceptor] on a project charter for a QI study. I read the charter myself. Great work! I am very impressed.”* *“Thank you! I look forward to working with you this coming month.* *“I heard about your interest in primary care too! Let's chat about it sometime in the next month!”* *“Sounds great!”*
*“teaching moment; vertigo, common presentation, can be scary given worry for posterior circulation stroke. important to distinguish central vs peripheral vertigo (impacted cerumen and external ear disease, BPPV, Labyrinthitis, vestibular neuronitis, Meniere’s ds.). would request the team to read up on the factors distinguishing the two. One thing to look up is HiNTs criteria/test. thank you”* *“[Dr. Preceptor], maybe you can [also] put in a good word with the powers that be...that we have little to no access to otoscopes. I couldn’t do a proper exam on this guy because i couldn’t find one. Wasn’t in the supply room.”*
*“[Dr. Preceptor] after reading this study, I think I would discuss the benefits and risks of follow up colonoscopy with the patient and perhaps not think it as important, since our patient had uncomplicated diverticulitis.”* *“I agree with you. Good work!!”*

## Discussion

Clinicians need efficient, effective, and responsible communication methods with both peers and trainees [13]. Previous reports have shown that most residents and staff prefer text messaging due to ease of use and efficiency [10, 14, 15]. Additionally, as we work as educators to balance patient privacy and autonomy with medical student inclusion in direct patient care, we are challenged to find innovative ways of using information technology to achieve these goals. Our mixed methods study suggests that integrated EMR secure messaging is a promising medium to enhance medical student team involvement as well as improve their education. Most medical students use personal smartphones for clinical work, and integrated secure messaging is an excellent alternative to other electronic messaging systems that may pose privacy concerns [5, 10, 13–15].

Our simple interventions in the current study resulted in students perceiving benefits to their education and involvement in patient care. Specifically, students reported a higher quality of communication with faculty and ancillary staff on their internal medicine rotation as compared to prior rotations. Furthermore, they noted that Secure Chat specifically contributed to improved quality and ease of communication on their internal medicine rotation, which was true for communication both with faculty and ancillary staff. Over 75% of students reported that Secure Chat improved their educational experience.

The review of message content provided insight into how students were being included in communications on Secure Chat. Over 50% of message threads contained information related to patient care, including daily management, admissions information, and discharge planning. These are conversations in which students may have not been directly included in the past. During acting internships, many students had robust message threads with attendings regarding patient management issues. Other interesting types of communications included a peer-to-peer communication regarding procedures as well as a communication from a resident highlighting a quality improvement issue.

The current study does have several limitations, including the partial survey format. Additionally, many students (7/24) had not worked on services with residents in the past, which could represent a significant confounder for perceived communication quality. The number of students involved in the study was also relatively low as we only selected one study site, and data collection prematurely ended due to the COVID-19 pandemic.

In future studies, additional interventions should be undertaken to promote use of integrated secure messaging with medical students. Specifically, more can be done to promote inclusion of medical students on secure messaging threads among faculty and residents. Direct integration into the clerkship could be enhanced through interventions such as weekly questions about specific cases and encouragement of peer-to-peer teaching. As integrated secure messaging becomes increasingly common and is adopted to a greater extent at institutions globally, faculty can work creatively to incorporate it as an additional tool for medical education and active student involvement in patient care.

## Conclusions

Integrated secure messaging systems present an opportunity to include students on important patient care conversations and decisions and to enhance their education. Our current mixed methods study suggests that students, faculty, and residents have high levels of satisfaction with utilization of Secure Chat and the impact it has on communication with team members and education. Additional efforts such as promotion with faculty and increased use in the clerkship or advanced electives can be further taken to promote use of integrated secure messaging and enhance student experiences.

## Data Availability

The datasets used and/or analyzed during the current study are available from the corresponding author on reasonable request.
